# Experience of Sorafenib as First-Line Treatment in Metastatic Renal Cell Carcinoma in a Tertiary Care Centre

**Published:** 2018-07-01

**Authors:** Mohith Saxena, Irappa Madabhavi, Apurva Patel, Harsha Panchal, Asha Anand

**Affiliations:** 1 Department of Medical and Pediatric Oncology, Gujarat Cancer Research Institute, Gujarat, Ahmedabad, India; 2 Department of Medical and Pediatric Oncology and Hematology, Kerudi Cancer Hospital, Bagalkot, Karnataka, India

**Keywords:** Renal cell carcinoma, Sorafenib, Metastatic, First line

## Abstract

**Background: **Metastatic renal cell carcinoma is chemoresistant and radioresistant disease with poor survival historically, but outcome has improved in past decade after introduction of tyrosine kinase inhibitors like sunitinib and sorafenib. Sorafenib has not been tested in Indian patients with metastatic RCC till now.

**Material and Methods: **This is a single arm, prospective, observational study done in unselected population of 60 patients with metastatic RCC treated with sorafenib as first- line therapy to assess efficacy and safety.

**Results: **Twenty three out of 60 patients (38.33%) continued sorafenib by the end of the study. Overall response rates (ORR), stable disease (SD) and disease control rates (DCR) were 35%, 43.33% and 78.33%, respectively. Median progression- free survival (PFS) and overall survival (OS) were 6 and 8 months, respectively and associated with histopathology, Memorial Sloan Kettering Cancer Centre (MSKCC) risk groups, Heng risk groups and performance status. Best tolerated dose was 400 mg per day which was half of standard dose. Fatigue, diarrhea, rashes and hand foot syndrome were common side effects while hypertension was rare.

**Conclusion: **Sorafenib, as first-line therapy, is an effective and safe treatment in Indian patients with metastatic RCC with poor tolerance to dose more than 400 mg per day. Side effects are mostly manageable.

## Introduction

 Renal cell carcinoma (RCC) accounts for 3% of adult malignancies globally^1^ with 5 year survival in early stage as high as 66%^2^. However, 5-year survival for the 30% patients who present with advanced and metastatic disease^3^ and another 25% patients who undergo localized resection and relapse with metastases^5^, is less than 10%^3^. Chemotherapy is not effective and cytokine therapy with interleukins or interferon-alfa produce modest response at the cost of significant toxicities in metastatic RCC^5^^-^^7^. 

Prognosis of metastatic RCC has improved significantly in recent time due to understanding of its molecular pathways. Von-Hippel-Landau (VHL) gene, a tumor suppressor gene which is regulator of platelet-derived growth factor (PDGF), vascular endothelial growth factor (VEGF), and other hypoxia-inducible factors (HIF) are found to be deleted, mutated, or altered in up to 80% of the patients with clear cell carcinoma, the most common subtype of RCC accounting for more than 80% of cases^8^^,^^9^. 

Sorafenib is a multikinase inhibitor which inhibits tumor proliferation and angiogenesis by inhibiting vascular endothelial growth factor receptors (VEGFR) 1, 2, and 3, platelet-derived growth factor receptor β (PDGFRβ); FMS-like tyrosine kinase 3 (Flt-3) c-Kit protein (c-Kit), Raf and RET receptor tyrosine kinases^10^. In placebo controlled trials, sorafenib has shown to improve progression-free survival (PFS) versus placebo in the treatment of naive patients and improvement both PFS and overall survival (OS) in treatment refractory patients^11^^,^^12^. It is relatively cheap, so suitable for Indian patients. Sunitinib and pazopanib, the other tyrosine kinase inhibitors which have shown to improve progression-free survival in phase III trials in patients with metastatic RCC as first-line therapy are beyond reach of most patients at our institute because of financial constraints^13^^,^^14^. Moreover, recent analysis showed no significant difference in PFS and OS between sorafenib and sunitinib as first-line therapy^15^^,^^16^. Data regarding use of tyrosine kinase inhibitors in Indian patients with metastatic RCC is sparse and sorafenib was never tested before among Indian patients with metastatic RCC to our best knowledge. So, this single arm, prospective, observational study was conducted at Gujarat Cancer & Research Institute to determine efficacy and safety of sorafenib among Indian patients with metastatic RCC.

## MATERIALS AND METHODS

**Study design**

This is prospective, single arm, single centre, observational study done at Gujarat Cancer & Research Institute over a period of three and half years (42 months) from January 2013 to June 2016.

The study included patients at least 18 years of age with histologically confirmed metastatic renal-cell carcinoma (RCC) with adequate bone marrow, liver, pancreatic, and renal function treated with sorafenib as first-line treatment. Patients with performance status within the range of the Eastern Cooperative Oncology Group (ECOG) criteria also entered the study.

**Dose modifications**

Starting dose of sorafenib was determined based on performance status, comorbidity and biochemistry profile. Doses were delayed or reduced if patients had clinically significant adverse events that were related to sorafenib. In such cases, doses were reduced to 400 mg once daily, and then to 200 mg once daily. If further reductions were required, sorafenib was permanently stopped. If adverse events resolved to a grade of 1 or less, the dose could be escalated to the previous level. 

**Baseline evaluation**

 This included medical history and physical examination, tumor imaging with computed tomography (CT) or magnetic resonance imaging (MRI) scans of the chest, abdomen and pelvis as well as bone scan, assessment of ECOG performance status, laboratory measurements (hematology, biochemistry including renal function and liver function, urinalysis, calcium and lactate dehydrogenase), cardiac function with electrocardiogram and two- dimensional echocardiography. 

**Primary end point**

Progression-free survival (PFS) was calculated as the time between the start of therapy and the date of progression or death from any cause.

**Secondary end point**

Objective response rate (ORR), overall survival (OS) and safety. ORR defined as the proportion of patients with confirmed complete response (CR) or partial response (PR). Clinical response {CR, PR, stable disease (SD)} and progressive disease (PD) were assessed according to response evaluation criteria in solid tumors (RECIST) using CT scans, MRI and bone scans (if bone metastases were present at baseline). Evaluations were done at regular intervals usually every 2 to 4 months. OS was calculated as the time between the start of therapy and the date of death due to any cause.

Toxicities were documented using the National Cancer Institute–Common Toxicity Criteria version 4.0 (NCI-CTC v4; Bethesda, MD).

**Statistical analysis**

Data were analysed using SPSS. Survival was calculated using Kaplan-Meir method.

## Results

 Between January 2013 and June 2015, a total of 70 patients with metastatic RCC were included in the study. All study participants received sorafenib and followed- up for a minimum of one year. Ten patients were lost at follow up and were therefore subsequently excluded from the study.

**Baseline characteristics**

The Median age was 55 years. Male to female ratio was 1.73:1. Most of patients in this study have ECOG performance status 2 (51.67%). Most common sites of metastasis were lung (66.67%) followed by bone (36.67%) and liver (20%). There was 100% concordance between Memorial Sloan Kettering Cancer Centre (MSKCC) risk groups^18^ and Heng risk groups^19^. Based on these prognostic schemes, seven patients (11.67%) with no risk factor were in favourable risk group, twenty-nine patients (48.33%) with one or two risk factors in intermediate risk group and twenty-four patients (40%) with three or more risk factors in poor risk group. (Table1)

**Table-1:** Baseline characterstics

**Characterstics**	**Number (%)**
Age	
31-40 yrs	8 (13.33)
41-50 yrs	15 (25)
51-60 yrs	23 (38.33)
61-70 yrs	13 (21.67)
>70 yrs	1 (1.67)
Sex	
Male	38 (63.33)
Female	22 (36.67)
ECOG-PS	
1	21 (35)
2	31 (51.67)
3	8 (13.33)
Site of metastases	
Lung	40 (66.67)
Bone	22 (36.67)
Liver	12 (20)
Soft tissue	11 (18.33)
Peritoneal	4 (6.67)
Brain	2 (3.33)
Adrenal	2 (3.33)
Ovarian	1 (1.67)
No. of metastases	
1	1 (1.67)
2	2 (3.33)
≥3	57 (95)
Histopathology	
Clear	50 (83.33)
Chromophobe	5 (8.33)
Papillary	4 (6.67)
Collecting duct	1 (1.67)
No. of MSKCC risk factors	
0 (Favorable)	7 (11.67)
1-2 (Intermediate)	29 (48.33)
≥3 (Poor)	24 (40)
No. of Heng risk factors	
0 (Favorable)	7 (11.67)
1-2 (Intermediate)	29 (48.33)
≥3 (Poor)	24 (40)

 Patients with ECOG performance status 1 without comorbidity and good nutritional status were started with 600 mg of sorafenib once daily, while patients with ECOG performance status 1 with comorbidity or poor nutritional status and ECOG performance status 2 without comorbidity and good nutritional status were started on 400 mg of sorafenib once daily. Sorfenib in dose of 200 mg once daily was offered as starting dose to patients with ECOG performance status 2 with comorbidity or poor nutritional status and ECOG performance status 3. Among the 60 evaluable patients, starting dose of sorafenib was 600 mg in 11 patients (18.33%), 400 mg in 39 patients (65%) and 200 mg in 10 patients (16.33%).

**Efficacy**

In this study, the median PFS with sorafenib was 6 months (range: 0.5 to 27 months), and the median OS was 8 months (range of 0.5 to 42 months). ORR (CR+PR) with sorafenib as first-line was 35% (CR=0%, PR=35%), and disease control rate (DCR=CR+PR+SD) was 78.33% (SD=43.33%). One- year PFS was 23.33%, and one-year OS was 43.33% in this study. (Table 2)

**Table 2:** Efficacy of sorafenib in renal cell carcinoma as first line

CR	PR	ORR	SD	CBR	Median PFS	1 year PFS	Median OS	1 year OS
0%	35%	35%	43.33%	78.33%	6 months	23.33%	8 months	43.33%

**Figure 1:**
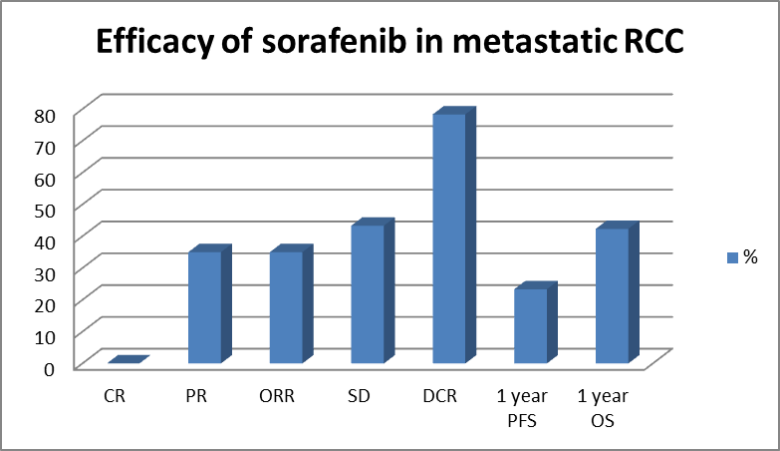
Efficacy of sorafenib in metastatic Renal Cell Carcinoma

**Figure 2:**
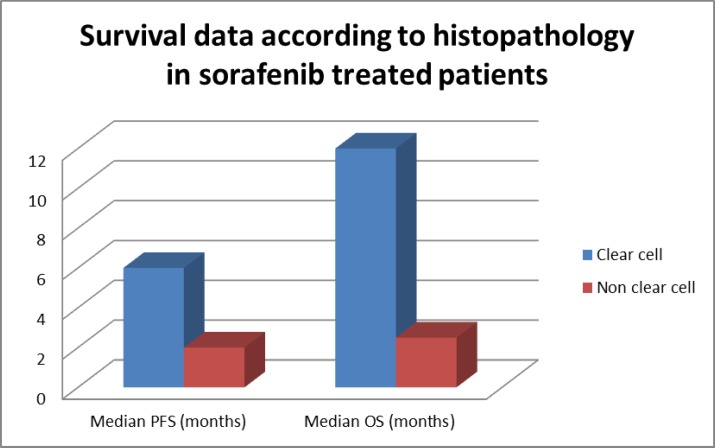
Survival data according to histopathology in sorafenib treated patients

**Table 3:** Median PFS & OS according to histopathology

**Histopathology**	**PFS**		**OS**	
	**Median**	**P value**	**Median**	**P value**
Clear cell	6	0.00576 (S)	12	0.01458
Non clear cell	2	-	2.5	-

**Table 4:** Median PFS & OS according to prognostic schemes

**MSKCC or Heng ** **prognostic ** **groups**	**Risk** **group**	**PFS**		**OS**	
		**Median**	**P value**	**Median**	**P value**
Favourable risk	0	20	0.00001 (S)	36	0.00001 (S)
Intermediate risk	1-2	8	0.00001 (S)	12	0.00001 (S)
Poor risk	≥ 3	2	-	2	

**Figure 3:**
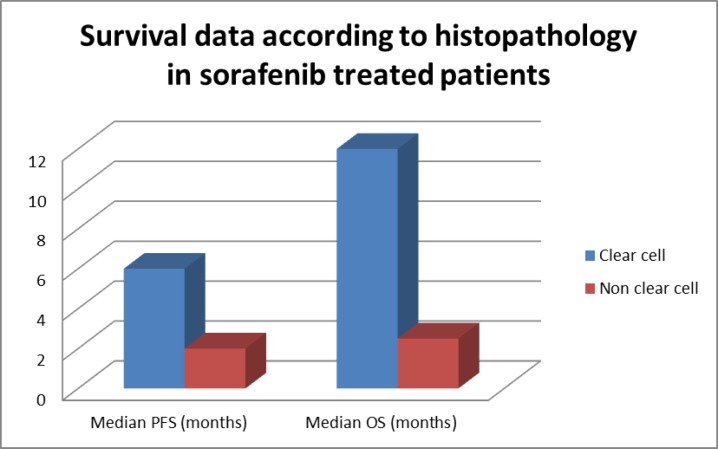
Survival data according to prognostic schemes in sorafenib treated patients

The median PFS and OS were significantly longer in patients with clear cell carcinoma than patients with non-clear cell carcinoma histology (Table 3, Figure 2). Median PFS and OS were significantly longer in patients with favourable risk and intermediate risk in comparison with poor risk patients (Table 4, Figure 3). Patients with ECOG performance status 1 had significantly longer OS in comparison to patients with ECOG performance status 2 and longer PFS and OS in comparison to patients with ECOG performance status 3 (Table 5).

**Table 5:** Median PFS & OS according to ECOG performance status

**ECOG** **Performance status**	**PFS**		**OS**	
	**Median**	**P value**	**Median**	**P value**
1	11	-	12	-
2	5	0.08585 (NS)	8	0.00696 (S)
3	2	0.00664 (S)	2	0.00899 (S)

**Safety**

**Figure 4:**
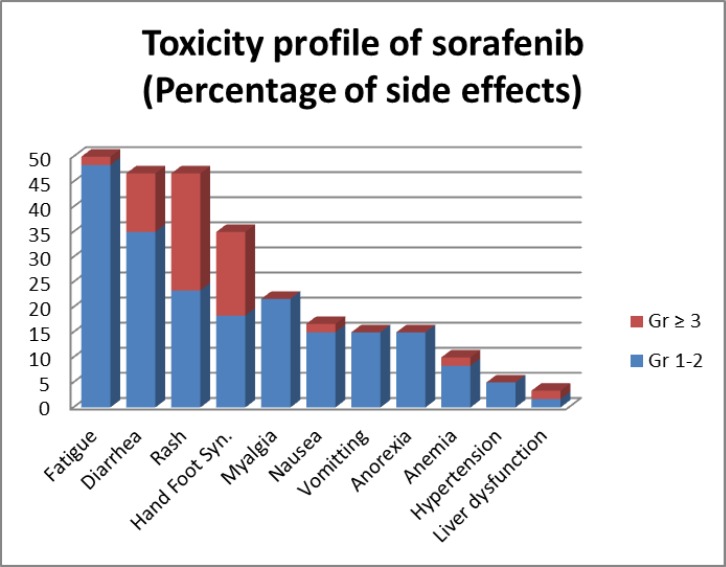
Toxicity profile of sorafenib

Toxicity profile of sorafenib is shown in Table 6 and Figure 4. Most common toxicities related to sorafenib were fatigue (50%) followed by diarrhoea (46.67%), rash (46.67%), hand foot syndrome (35%) and myalgia (21.67%). The most common grade 3 or higher toxicities related to sorafenib requiring dose modifications were rash (23.33%), hand foot syndrome (16.67%) and diarrhoea (11.67%). Two toxicity-related deaths were seen in the study: one was related to fulminant hepatic failure and the other one was related to severe diarrhea and mucositis; both were seen in the patient receiving 600 mg daily dose of sorafenib.

**Table 6:** Toxicity profile of sorafenib

**Toxicity**	**All Grade**		**Grade 1-2**		**Grade ≥ 3**	
	**No.**	**%**	**No.**	**%**	**No.**	**%**
Fatigue	30	50	29	48.33	1	1.67
Diarrhea	28	46.67	21	35	7	11.67
Rash	28	46.67	14	23.33	14	23.33
Hand Foot Syn.	21	35	11	18.33	10	16.67
Myalgia	13	21.67	13	21.67	0	0
Nausea	10	16.67	9	15	1	1.67
Vomitting	9	15	9	15	0	0
Anorexia	9	15	9	15	0	0
Anemia	6	10	5	8.33	1	1.67
Hypertension	3	5	3	5	0	0
Liver dysfunction	2	3.33	1	1.67	1	1.67

Discontinuation and Dose modifications: Twenty- three out of 60 patients (38.33%) were continued with sorafenib at the end of study. Among the remaining 47 patients (61.67%), the reason for discontinuation was progression in majority of patients (44 out of 47 patients, 93.62%). Only 3 patients (6.38%) discontinued sorafenib due to tolerance issue. Patients had very poor tolerance to 600 mg daily dose of sorafenib. Among the 11 patients treated with 600 mg daily dose of sorafenib, two toxic deaths (18.18%) were seen and 8 patients (72.72%) required dose reduction to 400 mg once daily mostly due to rashes and hand foot syndrome. Dose reductions were not required in patients treated with daily dose of 400 mg and 200 mg and no toxic death was noted in these patients. Dose escalations from starting dose were never possible due to tolerance issues.

## Discussion

 Treatment of metastatic RCC was challenging in the past as chemotherapy and radiotherapy were not much effective and cytokine therapy with interleukin-2 or interferon alfa had limited efficacy and considerable toxic effects^3^^,^^4^^,^^6^^,^^7^. With increase understanding of its molecular pathway and realizing importance of VHL gene, tyrosine kinase inhibitors were developed for treatment of metastatic RCC^8^^,^^9^.

Sorafenib was the first tyrosine kinase inhibitor which was approved by the United States Food and Drug Administration (US-FDA) in December 2005. It was considered as monotherapy by Ratain MJ et al for advanced RCC based on phase II trial in untreated patients of metastatic RCC. The results found an ORR of 36% (73 out of 202 patients) and SD of 32 % (65 out of 202 patients) with significant PFS advantage of 24 weeks for sorafenib versus 6 weeks for placebo in the 65 patients with stable disease at 12 weeks postrandomization^11^^.^ These results were comparable to our study.

 A subsequent phase III, randomized, placebo controlled trial of sorafenib in dose of 400 mg twice daily demonstrated significant PFS advantage of 5.5 months in sorafenib group versus 2.8 months in placebo group in treatment refractory patients of metastatic RCC^12^. The median OS was 19.3 months in sorafenib group and 15.9 months in placebo group which did not reach prespecified boundaries for statistical significance.

First-line therapy with sorafenib was compared with interferon alfa-2a and it was found that both treatments were similar (5.7 months versus 5.6 months) with regard to PFS, but patients treated with sorafenib had greater tumor shrinkage (68.2% versus 39%), better quality of life and improved tolerability^20^. CR, PR and disease control rates (DCR) were 0%, 5.2% and 79.4%, respectively for sorfenib versus 1.1%, 7.6% and 64.1%, respectively for interferon alfa-2a. Only patients with ECOG performance status 0 and 1 and clear cell histology were included. In our study, we found almost similar PFS, but better PR and DCR with sorafenib in unselected patients with metastatic RCC. In a recently published PREDICT study performed on unselected patients of metastatic RCC, ORR and DCR were 23.4% and 70.4%, respectively overall and 31.4% and 94.6%, respectively after excluding patients with no radiological assessment^21^. The median PFS was 7.6 months in patients with no prior therapy and 7.1 months in patients who received one or more prior therapies. These results are similar to those obtained in our study.

Sorafenib was found to be effective in Asian patients as well. In a Chinese study, CR, PR, ORR, SD, DCR median PFS and 1-year PFS were 1%, 23.5%, 24.5%, 63.3%, 87.8%, 60 weeks and 58.4%, respectively, while in a Korean study, ORR, DCR and median PFS in patients with metastatic RCC treated with with sorafenib as first-line therapy were 23.2%, 56% and 7.4 months, respectively. PFS data in these studies were better than those of our study^22^^,^^23^. 

Sorafenib is useful first-line therapy even after introduction of sunitinib in treatment of metastatic RCC. Though an indirect comparison meta-analysis of 6 trials showed superiority of sunitinib over sorafenib and bevacizumab plus interferon alfa, a Korean study and a retrospective analysis in Chinese patients reported no difference in PFS and OS between sunitinib and sorafenib as first-line therapy^24^^,^^25^^,^^16^. 

Data regarding the use of tyrosine kinase inhibitors are sparse. Only sunitinib was tested in Indian patients with PFS of 11.4 months in a study from Tata Memorial Hospital and PFS of 7.5 months in another study from our institute^25^^, ^^26^. This is the first study establishing efficacy and safety of sorafenib in Indian patients with metastatic RCC. Further studies are required to compare sorafenib with sunitinib as first-line treatment in Indian patients. We also validated prognostic schemes by MSKCC and Heng in Indian patients^18^^, ^^19^.

In this study, it was shown that Indian patiets have very poor tolerance to standard dose of sorafenib. No patient in our study could be escalated to standard dose of 800 mg per day and 72.72% receiving dose of 600 mg per day required dose reduction with two toxic deaths (18.18%). However, patients receiving 400 mg or 200 mg daily dose tolerated well with no dose reduction or toxic death in these groups. Four hundred milligram daily dose was best tolerated dose of sorafenib in this study which was half of that given in the above- mentioned study. Still, our results were comparable to most of studies. Most common toxicities related to sorafenib were fatigue followed by diarrhoea, rash, hand foot syndrome and myalgia. The most common grade 3 or higher toxicities related to sorafenib requiring dose modifications were rash, hand foot syndrome and diarrhoea. Toxicity profile was different in this study. Our patients reported more fatigue, similar diarrhea, rash and hand foot syndrome and less hypertension than western patients^12^. This study showed higher incidence of fatigue, diarrhea, skin rashes and hand-foot syndrome but lower incidence of hypertension compared to Chinese and Korean studies^22^,^23^. Different polymorphisms in genetic profile and different metabolism might be responsible for this difference which needs to be further investigated.

## CONCLUSION

 Sorafenib is one of the several agents that target proangiogenic growth factor pathway in the pathogenesis of metastatic RCC and is shown clinical activity in clinical trials so it is a viable option. This is the first study establishing its safety and efficacy in Indian patients with comparable response rates and PFS to most of studies. In the present study, a half dose of Sorafenib was better tolerated by patients compared to other studies. Toxicity profile was different and most of the side effects were easily manageable. Careful patient selection, dose adjustment, counselling and follow- up are required to get optimal results.

## References

[1] Parkin DM, Bray F, Ferlay J (2005). Global cancer statistics, 2002. CA Cancer J Clin.

[2] Schoffski P, Dumez H, Clement P (2006). Emerging role of tyrosine kinase inhibitors in the treatment of advanced renal cell cancer: A review. Ann Oncol.

[3] Motzer RJ, Bander NH, Nanus DM (1996). Renal-cell carcinoma. N Engl J Med.

[4] Cohen HT, McGovern FJ (2005). Renal-cell carcinoma. N Engl J Med.

[5] Janzen NK, Kim HL, Flglin RA (2003). Surveillance after radical or partial nephrectomy for localized renal cell carcinoma and management of recurrent disease. Urol Clin North Am.

[6] Law TM, Motzer RJ, Mazumdar M (1995). Phase III randomized trial of interleukin-2 with or without lymphokine-activated killer cells in the treatment of patients with advanced renal cell carcinoma. Cancer.

[7] Negrier S, Escudier B, Lasset C (1998). Recombinant human interleukin-2, recombinant human interferon alfa-2a, or both in metastatic renal-cell carcinoma. Groupe Français d′Immunothérapie. N Engl J Med.

[8] Na X, Wu G, Ryan CK (2003). Overproduction of vascular endothelial growth factor related to von Hippel-Lindau tumor suppressor gene mutations and hypoxia-inducible factor-1 alpha expression in renal cell carcinomas. J Urol.

[9] Maxwell PH, Wiesener MS, Chang GW (1999). The tumour suppressor protein VHL targets hypoxia-inducible factors for oxygen-dependent proteolysis. Nature.

[10] Wilhelm SM, Carter C, Tang L (2004). BAY 43-9006 exhibits broad spectrum antitumor activity and targets the Raf/MEK/ERK pathway and receptor tyrosine kinases involved in tumor progression and angiogenesis. Cancer Res.

[11] Ratain MJ, Eisen T, Stadler WM (2006). Phase II placebo-controlled randomized discontinuation trial of sorafenib in patients with metastatic renal cell carcinoma. J Clin Oncol.

[12] Escudier B, Eisen T, Stadler WM (2007). Sorafenib in advanced clear-cell renal-cell carcinoma. N Engl J Med.

[13] Motzer RJ, Hutson TE, Tomczak P (2007). Sunitinib versus interferon alfa in metastatic renal-cell carcinoma. N Engl J Med.

[14] Sternberg CN, Davis ID, Mardiak J (2010). Pazopanib in locally advanced or metastatic renal cell carcinoma: results of a randomized phase III trial. J Clin Oncol.

[15] Park SJ, Lee JL, Park I (2012). Comparative efficacy of sunitinib versus sorafenib as first-line treatment for patients with metastatic renal cell carcinoma. Chemotherapy.

[16] Sheng X, Chi Z, Cui C (2016). Efficacy and safety of sorafenib versus sunitinib as first-line treatment in patients with metastatic renal cell carcinoma: largest single-center retrospective analysis. Ontarget.

[17] National Cancer Institute (2010). Common Terminology Criteria's for Adverse Events Version 4.03.

[18] Motzer RJ, Bacik J, Murphy BA (2002). Interferon-alfa as a comparative treatment for clinical trials of new therapies against advanced renal cell carcinoma. J Clin Oncol.

[19] Heng DY, Xie W, Regan MM (2009). Prognostic factors for overall survival in patients with metastatic renal cell carcinoma treated with vascular endothelial growth factor-targeted agents: Results from a large, multicentre study. J clin Oncol.

[20] Escudier B, Szczylik C, Hutson TE (2009). Randomized phase II trial of first-line treatment with sorafenib versus interferon alfa-2a in patients with metastatic renal cell carcinoma. J Clin Oncol.

[21] Jager D, Ma JH, Mardiak J (2015). Sorafenib treatment of advanced renal cell carcinoma patients in daily practice: The large international PREDICT study. Clin Genitourin Cancer.

[22] Zhang H, Dong B, Lu JJ (2009). Efficacy of sorafenib on metastatic renal cell carcinoma in Asian patients: Results from a multicentre study. BMC Cancer.

[23] Kim SH, Kim S, Nam BH (2015). Efficacy and safety of sorafenib therapy on metastatic renal cell carcinoma in Korean patients: Results from a retrospective multicentre study. PLoS One.

[24] Mills EJ, Rachlis B, O′Regan C (2009). Metastatic renal cell cancer treatments: An indirect comparison meta-analysis. BMC Cancer.

[25] Krishna VM, Noronha V, Prabhash K (2013). Sunitinib in metastatic renal cell carcimoma: A single-center experience. Indian J Cancer.

[26] Patel KB, Panchal HP, Karanwal AB, Parekh BB, Shah S, Prasad S (2016). Sunitinib in metastatic renal cell carcinoma: Experience from single centre study, efficacy and safety. Indian J Cancer.

